# Atypical case report of hepatocellular carcinoma mimicking gallbladder cholangiocarcinoma: A diagnostic challenge

**DOI:** 10.1016/j.ijscr.2025.111928

**Published:** 2025-09-10

**Authors:** Mohamed Ali Chaouch, Mohamed Zayati, Ibtissem Korbi, Midani Touati, Ramzi Beltaifa, Faouzi Noomen

**Affiliations:** a Department of Visceral and Digestive Surgery, Monastir University Hospital, Tunisia; b Department of Anesthesiology, Monastir University Hospital, Tunisia

**Keywords:** Hepatocellular carcinoma, Cholangiocarcinoma, Gallbladder cancer, Histopathology, Liver malignancy, Diagnostic imaging, Case report

## Abstract

Introduction and importance: Hepatocellular carcinoma (HCC) and cholangiocarcinoma (CCA) are the two most common primary hepatic malignancies of distinct origins, but they can present with overlapping clinical and imaging features. Accurate differentiation is crucial, especially as treatment and prognosis differ significantly. This report presents a rare case of HCC mimicking gallbladder CCA, highlighting diagnostic challenges.

**Case presentation:**

A 63-year-old man with diabetes and hypertension presented with progressive jaundice, fatigue, and weight loss. Imaging through CT and magnetic resonance imaging revealed a gallbladder mass that invaded the liver, suggesting CCA of the gallbladder. Surgical exploration and en bloc resection were performed, including bisegmentectomy (IVb and V), cholecystectomy, and lymphadenectomy. Postoperative histopathology revealed a lymphocyte-rich and well-differentiated variant of HCC, not CCA.

**Clinical discussion:**

Despite the imaging and clinical indications of CCA, histological analysis confirmed the presence of HCC. The case highlights the limitations of imaging in atypical presentations and underscores the value of biopsy in obtaining a definitive diagnosis. The accurate classification of liver malignancies is critical for determining therapeutic strategies, such as eligibility for liver transplantation, and for prognostic accuracy.

**Conclusion:**

This case highlights the diagnostic challenge of distinguishing HCC from CCA when imaging features overlap. Histopathological confirmation remains essential, and a multidisciplinary approach is vital for optimal patient management.

## Highlights

• This case demonstrates an atypical presentation of hepatocellular carcinoma (HCC) that radiologically and clinically mimicked gallbladder cholangiocarcinoma (CCA), leading to diagnostic confusion.
• Despite comprehensive imaging with CT and MRI, the diagnosis remained inconclusive preoperatively, underscoring the limitations of radiological techniques in differentiating hepatic malignancies in atypical scenarios.
• The final diagnosis of a well-differentiated, lymphocyte-rich variant of HCC was only established after surgical resection and histopathological examination, highlighting the necessity of tissue diagnosis to guide appropriate management.

## 1 Introduction

Hepatocellular carcinoma (HCC) and cholangiocarcinoma (CCA) are the two most common primary hepatic malignancies [1]. CCA, though less common, is the second most common primary hepatic malignancy and originates in the biliary epithelium, most often presenting as an adenocarcinoma [2]. Although they differ in origin and management, both can present with overlapping clinical and imaging features, which complicates diagnosis and treatment planning [3]. This report, according to the SCARE guidelines [4], describes a rare case of HCC mimicking gallbladder CCA, diagnosed only after surgical resection and histopathological analysis, highlighting the limitations of imaging and the importance of biopsy.

## 2 Case presentation

**Fig. 1:**
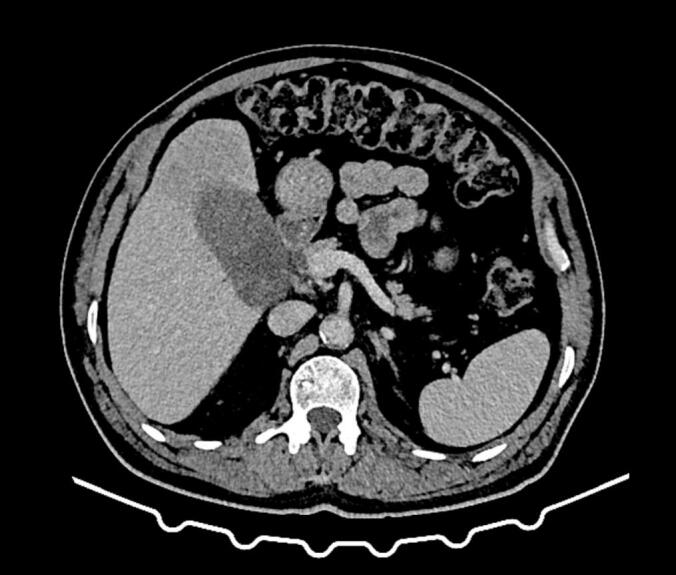
Contrast-enhanced axial CT scan demonstrating a mass involving the gallbladder fundus with contiguous extension into adjacent liver parenchyma (segment IV), suggestive of gallbladder malignancy.

**Fig. 2:**
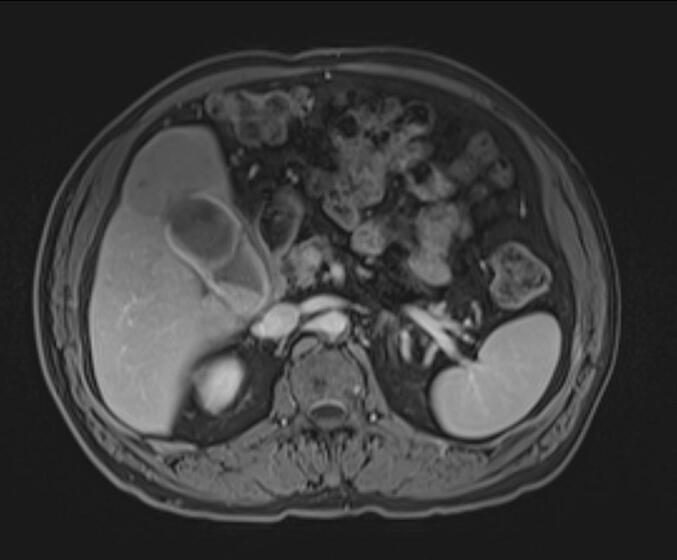
Axial T1-weighted contrast-enhanced MRI showing a heterogeneous gallbladder mass with liver invasion (segments IV and V), radiologically mimicking gallbladder cholangiocarcinoma.

**Fig. 3:**
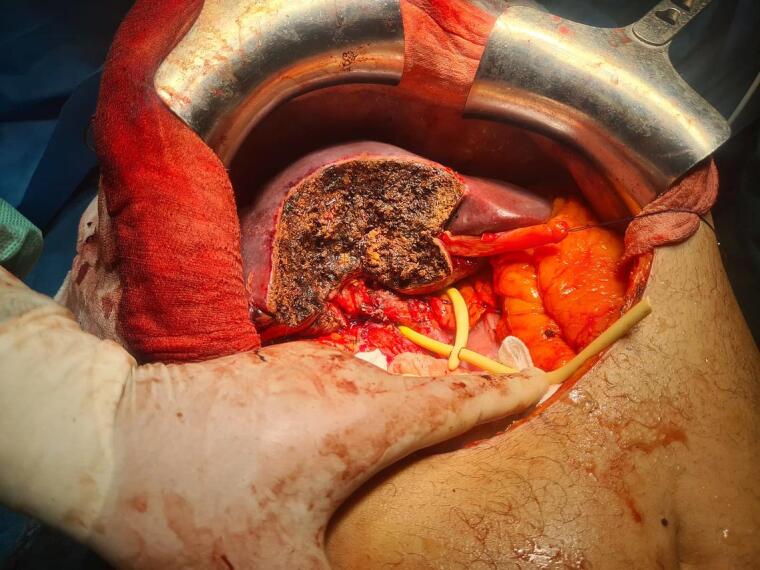
Intraoperative image showing the liver after bisegmentectomy (segments IVb and V), revealing the resected tumor bed with clear demarcation between normal and involved liver tissue.

We present the case of a 63-year-old male patient with a medical history of type 2 diabetes mellitus and hypertension. He presented with two months of progressive jaundice, fatigue, and weight loss. Physical examination revealed a marked icterus. The abdomen was soft and non-tender, without palpable masses or hepatomegaly; the gallbladder was not palpable. Laboratory investigations showed preserved liver function with normal bilirubin and transaminases, but mild elevation of alkaline phosphatase and γ-glutamyl transferase, consistent with biliary obstruction. Serum alpha-fetoprotein (AFP) level was within normal limits, while CA 19-9 was mildly elevated. Viral hepatitis serology (HBsAg and anti-HCV) was negative. Initial abdominal CT revealed a suspicious mass located in the fundus of the gallbladder with signs of direct invasion into segment IV of the liver (Fig. 1). A second lesion, nodular in appearance and measuring 18 mm, was identified in the infundibulum of the gallbladder that extends into the cystic duct. The perivesical fat was infiltrated and the mass was near the right colic flexure, although a thin fat plane was preserved, indicating no gross invasion. To further characterize the lesion, contrast-enhanced magnetic resonance imaging (MRI) of the liver was performed. Magnetic resonance imaging demonstrated a malignant-appearing gallbladder mass involving the corpus-fundic region, with contiguous extension into liver segments IV and V (Fig. 2). These imaging findings, along with the clinical presentation, suggest a gallbladder cholangiocarcinoma. A thoracic CT scan was performed for staging, which showed no evidence of distant metastasis. The patient was taken to surgery via a Makuuchi incision. Intraoperatively, a tumor was observed involving the gallbladder and infiltrating segment IV of the liver, without evidence of peritoneal or liver metastases. An en bloc resection was performed, including a bisegmentectomy of the IVb and V with cholecystectomy and regional lymphadenectomy along the common hepatic artery (Fig. 3). The postoperative course was uneventful. Histopathological examination of the surgical specimen revealed a well-differentiated hepatocellular carcinoma of the lymphocyte-rich variant. The neoplastic cells showed strong positivity for HepPar-1, Arginase-1, and Glypican-3, and were negative for CK7 and CK19, excluding a biliary phenotype. The lymphocytic infiltrate consisted predominantly of CD3-positive T-cells, consistent with the lymphocyte-rich variant. The tumor infiltrated the gallbladder wall and extended close to the serosa, within a 1 mm margin. There was no histological evidence of cholangiocarcinoma. The patient was followed for six months postoperatively with regular clinical evaluation and imaging. At the last follow-up, he remained asymptomatic, with no evidence of disease recurrence or metastasis. Liver function tests were within normal limits, and his overall recovery was satisfactory.

## 3 Discussion

CCA is a malignant epithelial tumor arising from the epithelium of the bile duct, and it can occur in intrahepatic, perihilar, or distal extrahepatic locations [1]. It is histologically characterized as an adenocarcinoma and represents the second most common primary hepatic malignancy after HCC. Although HCC arises from hepatocytes and is often associated with chronic liver disease, CCA occurs more frequently de novo or in association with conditions such as primary sclerosing cholangitis, hepatolithiasis, or parasitic infections [5]. Despite their distinct origins and biological behaviors, HCC and CCA may present with overlapping clinical features, such as jaundice, weight loss, and nonspecific abdominal discomfort. The challenge in differentiation becomes especially pronounced in atypical presentations, as illustrated in our case, in which an HCC mimicked a gallbladder CCA based on imaging characteristics [6]. In our patient, the imaging findings were atypical for HCC. Classically, HCC demonstrates intense arterial phase hyperenhancement with rapid washout in the portal venous or delayed phases and may show a capsule appearance on MRI. By contrast, CCA usually appears hypovascular, with gradual peripheral-to-central enhancement. In this case, the mass presented as a gallbladder-centered lesion with contiguous extension into segments IV and V of the liver, closely resembling gallbladder CCA both morphologically and by its infiltrative pattern. The absence of classic HCC features, such as distinct washout and capsule formation, contributed to the misinterpretation. Similar diagnostic dilemmas have been reported in the literature, where atypical HCCs, particularly the lymphocyte-rich and well-differentiated variants, exhibit imaging overlap with CCA, leading to diagnostic uncertainty. Radiologists have emphasized that up to 6–10 % of CCAs may mimic HCC with early enhancement patterns, while conversely, atypical HCCs may lack classical enhancement behavior, underscoring the importance of histological verification [7]. Incorporating expert radiological opinions and literature supports the notion that even with multiphasic CT and dynamic MRI, such cases remain diagnostically challenging and highlight the need for a multidisciplinary approach.

Radiologically, CCA typically appears as a hypovascular mass with delayed peripheral contrast enhancement, while HCC is generally hypervascular during the arterial phase with washout in the portal venous phase [7]. However, there are exceptions to these imaging patterns. In some atypical cases, intrahepatic CCA or gallbladder CCA may demonstrate early arterial enhancement, mimicking the radiographic appearance of HCC. Studies have shown that up to 6 % of cholangiocarcinomas can exhibit such enhancement patterns, complicating radiological interpretation. Advanced imaging modalities such as multiphasic CT and dynamic contrast-enhanced magnetic resonance imaging are essential tools in preoperative evaluation [8]. However, as seen in this case, even with these techniques, a definitive diagnosis may remain elusive. In such cases, histopathological confirmation remains the gold standard. Mimicry between HCC and CCA, though uncommon, is documented in 5–10 % of cases. Radiologically, HCC may show a capsule, intratumoral fat, or nodular-in-nodular architecture, while CCA more often demonstrates biliary dilatation, capsular retraction, and delayed enhancement. Serologically, AFP is elevated in only ∼60 % of HCCs, and CA 19-9 or CEA may rise in CCA, but none are specific. Thus, while these markers may suggest one entity over the other, they are insufficient for definitive diagnosis, reinforcing the need for histopathology. Biopsy provides not only diagnostic clarity but also prognostic information, such as tumor grade and variant subtype, which are crucial to guide treatment decisions. In our case, a pre-operative biopsy was not pursued. The mass appeared gallbladder-centered with contiguous hepatic invasion, radiologically consistent with gallbladder CCA. Given the strong clinical and radiological suspicion, combined with the patient's good surgical fitness, upfront en bloc resection was favored as both a diagnostic and therapeutic procedure. Additionally, concerns about potential tumor seeding and the limited diagnostic yield of gallbladder biopsies in infiltrative masses further supported this decision. This highlights a practical limitation often faced in surgical oncology, where definitive histopathology is sometimes only established after resection. In our patients, the diagnosis of a lymphocyte-rich variant of HCC, a relatively rare subtype, was only made postoperatively by histological analysis. This distinction between HCC and CCA is particularly critical when considering treatment options [9]. For example, liver transplantation is a potentially curative option in selected cases of HCC but is contraindicated in CCA due to high recurrence rates. The lymphocyte-rich variant of HCC accounts for ∼1–2 % of cases and is associated with a relatively favorable prognosis due to the prominent immune response. In our patient, tumor cells stained positive for HepPar-1, Arginase-1, and Glypican-3, but negative for CK7/CK19, excluding CCA [6]. The dense CD3-positive T-cell infiltrate was consistent with reported cases. This rare subtype often presents with atypical imaging findings, reinforcing the diagnostic challenge. In addition, systemic therapies, resection strategies, and prognostic expectations differ significantly between the two entities. Our case underscores the importance of maintaining a broad differential diagnosis when evaluating liver and gallbladder masses and highlights the limitations of imaging in atypical presentations. A multidisciplinary approach that integrates imaging, surgical assessment, histopathology, and, where available, molecular profiling is essential for accurate diagnosis and optimal management.

## 4 Conclusions

This case underscores the importance of maintaining a broad differential diagnosis when evaluating hepatic and gallbladder masses. HCC can rarely mimic CCA both radiologically and clinically, creating significant diagnostic uncertainty. Neither imaging nor serology alone is reliable for distinction, particularly in atypical presentations. Histopathological confirmation remains the gold standard and should guide treatment planning. Clinicians should recognize this diagnostic pitfall, avoid over-reliance on imaging patterns, and ensure early multidisciplinary discussion to optimize outcomes.

## Author contribution

Hafedh Daly, Faiez Boughanmi, Mohamed Ali Chaouch, Midani Touati, Fethi Jebali, and Bahri Mahjoub participated in the manuscript and validated the final version of the manuscript.

## Consent

Written informed consent was obtained from the patient for publication and the accompanying images. A copy of the written consent is available for review by the Editor-in-Chief of this journal on request.

## Ethical approval

Ethical approval is exempt/waived at our institution.

## Guarantor

Mohamed Ali Chaouch, MD.

## Research registration number

N/A.

## Funding

This research did not receive a grant from the public, commercial or non-profit sectors.

## Conflict of interest statement

No conflict of interest to disclose.
